# Sustainability of Endovenous Iron Deficiency Anaemia Treatment: Hospital-Based Health Technology Assessment in IBD Patients

**DOI:** 10.1155/2017/3470893

**Published:** 2017-07-06

**Authors:** A. Poscia, J. Stojanovic, F. Kheiraoui, E. M. Proli, F. Scaldaferri, M. Volpe, M. L. Di Pietro, A. Gasbarrini, L. Fabrizio, S. Boccia, C. Favaretti

**Affiliations:** ^1^Section of Hygiene, Institute of Public Health, Università Cattolica del Sacro Cuore of Rome, Largo F. Vito 1, 00168 Rome, Italy; ^2^Hospital Pharmacy, Policlinico Umberto I, Viale del Policlinico 155, 00161 Rome, Italy; ^3^Department of Internal Medicine and Gastroenterology, Fondazione Policlinico A. Gemelli, Largo A. Gemelli 8, 00168 Rome, Italy; ^4^Hospital Pharmacy, Fondazione Policlinico A. Gemelli, Largo A. Gemelli 8, 00168 Rome, Italy; ^5^Hospital Health Management, Fondazione Policlinico A. Gemelli, Largo A. Gemelli 8, 00168 Rome, Italy; ^6^Section of Hygiene, Institute of Public Health, Università Cattolica del Sacro Cuore, Fondazione Policlinico A. Gemelli, Largo F. Vito 1, 00168 Rome, Italy

## Abstract

Iron deficiency anaemia (IDA) is the main extraintestinal manifestation affecting patients with inflammatory bowel disease (IBD). The Health Technology Assessment approach was applied to evaluate the sustainability of intravenous (IV) iron formulations in the Italian hospital setting, with particular focus on ferric carboxymaltose. Data on the epidemiology of IBD and associated IDA, in addition to the efficacy and safety of IV iron formulations currently used in Italy, were retrieved from scientific literature. A hospital-based cost-analysis of the outpatient delivery of IV iron treatments was performed. Organizational and ethical implications were discussed. IDA prevalence in IBD patients varies markedly from 9 to 73%. IV iron preparations were proven to have good efficacy and safety profiles, and ferric carboxymaltose provided a fast correction of haemoglobin and serum ferritin levels in iron-deficient patients. Despite a higher price, ferric carboxymaltose would confer a beneficial effect to the hospital, in terms of reduced cost related to individual patient management and additionally to the patient by reducing the number of infusions and admissions to healthcare facilities. Ethically, the evaluation is appropriate due to its efficacy and compliance. This assessment supports the introduction of ferric carboxymaltose in the Italian outpatient setting.

## 1. Introduction

Inflammatory bowel disease (IBD), which includes ulcerative colitis (UC), Crohn's disease (CD), or indeterminate colitis, is characterized by chronic relapsing inflammation of the bowel [1]. The aetiology of IBD is not fully understood but evidence indicates an immune dysfunction that is influenced by a genetic predisposition, the enteric microflora, and environmental triggers.

A significant proportion of IBD patients suffer from extraintestinal manifestations affecting different organs, with the most frequent one being anaemia [2]. The most prevalent IBD associated anaemia types are iron deficiency anaemia (IDA) and anaemia of chronic disease, with cases of vitamin B12 or folic acid deficiency, and drug-induced anaemia presenting infrequently [3]. Iron deficiency anaemia results from chronic blood loss in the gastrointestinal tract combined with the reduced uptake of iron by enterocytes due to inflammation. In contrast, anaemia of chronic disease is caused by chronic inflammation limiting the availability of iron for erythropoietic bone marrow cells through a cytokine mediated cascade and impeding their iron saturation [4].

Comorbid anaemia in IBD individuals is a significant predictor of an increased risk of hospitalization and even increased patient mortality [5]. A recent study by Koutroubakis et al. [6] identified that the patterns of anaemia in IBD patients are associated with aggressive and disabling disease over time. In this prospective cohort, study patients with IBD and persistent or recurrent anaemia required significantly more healthcare visits and had higher indices of disease activity, as well as a lower average quality of life, than nonanaemic patients.

When treated in line with WHO guidelines, iron supplementation should aim for a posttreatment haemoglobin (Hb) level of 130 g/l in men and 120 g/l in women [7]. For patients with a known intolerance or lack of response to oral iron, intravenous (IV) iron is considered a safe alternative [8], which can provide a fast and effective response. Despite IV iron alternatives being generally better tolerated by patients and having lower discontinuation rates [9], their general adoption is limited by the possibility of rare but potentially life-threatening side effects. Additionally, IV treatment may require additional staff or facilities which can limit its uptake. According to the ECCO guidelines [1], absolute indications for intravenous iron include severe anaemia (Hb < 10 g per 100 ml), extreme intestinal disease activity, concomitant therapy with an erythropoiesis-stimulating agent, or patient preference [10].

The release of new IV iron compounds represents a major advantage for both physicians and patients with IDA and may potentially improve the therapeutic algorithm [11, 12]. However, without appropriate multidimensional evaluations, the higher costs related to these technological innovations could withhold their optimal uptake into clinical practice. For this reason, Health Technology Assessment (HTA) plays an essential role in current healthcare systems by supporting evidence-based decision-making in healthcare policy and practice. HTA is concerned with the medical, organizational, economic, and societal consequences of using health technologies within the health system [13, 14] and focuses on the overall value of the technology in the current clinical practice [15, 16]. Hospital decision-makers remain in the front line of innovative technologies and need to be able to manage available resources more efficiently. Thus, they are increasingly requesting HTA evaluations tailored to their specific context.

For this reason, we applied the HTA approach to provide insights on the sustainability of the IV iron formulation in a hospital setting. Particularly, we placed a specific focus on the impact analysis of the introduction of ferric carboxymaltose in one outpatient gastroenterology department specifically dedicated to patients with IBD.

## 2. Methods

### 2.1. Disease Epidemiology

A scoping review was performed to identify recent evidence on the epidemiology of IBD and IDA associated with this condition. A bibliographic search in MEDLINE, an online database, was conducted and all relevant systematic reviews/reviews and meta-analyses published in English or Italian were assessed. The search terminology included all possible variations of the following terms: Crohn's disease, ulcerative colitis, IBD, and anaemia.

### 2.2. Efficacy Safety of IV Iron

A literature search was performed in the MEDLINE database to retrieve data on the efficacy and safety of IV iron formulations commonly used for IDA, with particular focus on the IBD population. Meta-analyses, systematic reviews, and randomized clinical trials (RCTs) were considered, with language being the only restriction used (English or Italian).

### 2.3. Organizational Aspects

With the aim to evaluate the methods of administration and the organizational aspects of the IV iron formulations in the hospital setting, we performed an analysis of the current literature, as well as a detailed assessment of the Summary of Product Characteristics of the therapeutic options currently available in Italy [17].

### 2.4. Economic Evaluation

A hospital-based cost-minimization analysis of the ambulatory delivery of IV iron treatments was performed. In the base model, we chose to compare the treatments currently available at “A. Gemelli” Foundation Polyclinic (Rome, Italy) taking into consideration a standard patient with an iron supplementation need of 1000 mg. The options considered were ferric gluconate (ferric gluconate®) and ferric carboxymaltose (ferric carboxymaltose®). In relation to the product characteristics, three possible modes of administration were analysed: ferric carboxymaltose 1000 mg (2 vials in a single session); ferric gluconate 1000 mg (1 vial per session—16 sessions); and ferric gluconate 1000 mg (2 vials per session—8 sessions).

To determine the overall cost of the treatment, we calculated the labour cost and the cost of acquisition, preparation, and disposal of the drug. Reimbursement rates for the respective treatments were based on the 2013 Italian National Health System ambulatory pricelist.

Mean salaries of nurses and physicians were obtained from the internal administrative database in compliance with the Italian National Collective Agreements. The hourly salary rate was calculated taking into consideration days of absence and holidays.

Personnel expenses were determined by multiplying the time required for delivery of therapy (patient admission, obtaining consent, preparing the site of administration, drug infusion, and detachment) with the hourly cost of dedicated personnel. For both treatments, based on expert guidelines, we included a medical examination before the first treatment. In the case of ferric gluconate, a “subsequent visit” before the next one was included, given the long period between the first and the last infusion. The determination of the time required for the delivery of therapy and the amount of used materials was estimated by direct observation of the gastroenterology department, in accordance with the patient and personnel safety procedures.

The medication cost used in the base model was derived from the authorization report of the Italian regulatory authority (AIFA) with prices of 91.00 € and 4.36 € for ferric carboxymaltose (500 mg) and ferric gluconate (312.5 mg), respectively.

The costs associated with drug preparation (including disposable materials—saline, syringes, infusion kits, and disposable gloves) and its disposal were obtained from an internal administrative database. The amount of used materials was estimated by direct observation of the gastroenterology department, in accordance with the patient and personnel safety procedures. The cost of disposal was estimated as a product of the weight of the waste for the average tariff applied to the disposal of hospital waste in the Lazio region [18]. The weight of waste was calculated at the end of a transaction carried out at the outpatient gastroenterology clinic.

A sensitivity analysis was conducted to define the plausibility of the costs of the treatments under review. The variables used to construct the possible scenarios were the time that staff dedicated to an infusion (time of administration) and the cost of the drug, due to their high impact on the overall cost of therapy.

The plausibility range was determined based on the considerations derived from the literature review performed in the section related to organizational aspects. Consequently, in the sensitivity analysis, we assumed that the minimum time for both treatments was the average time estimated by experts (the amount of time sufficient to guarantee patient safety). In contrast, the maximum value for both treatments was considered to be the overall time required for the infusion of the drugs.

However, since the costs of intravenous iron formulations are influenced by the choice of agent, the number of treatments, and consequently specific price agreements with the pharmaceutical companies, in the sensitivity analysis, the costs of 1000 mg therapy ranged from 85.26 € to 271.10 € and from 7.36 € to 20.80 € for ferric carboxymaltose and ferric gluconate, respectively.

Moreover, given the variability in each patient's clinical need for supplementation, the sensitivity analysis was conducted using a standard patient, as used in the basic model (1000 mg of iron), in addition to a patient with a 500 mg requirement.

### 2.5. Ethical Evaluation

Unlike the other fields of a HTA process, which are based on standardized methods of analysis, the ethical assessment is influenced not only by efficacy, tolerability, safety, and economics, but also by the philosophical foundation and the methodology of analysis. In this assessment, the philosophical approach is cognitivist and focuses on the human being* (person-centred ethics)*. The morality of an act, therefore, depends on the respect for each human being's life, health, and autonomy. Based on a person-centred approach, this ethical evaluation includes three phases: review of the available evidence on the specific technology and the assessment of the proportionality risks/benefits* (epistemological moment)*; analysis of this data in light of the references ethics, to understand whether or not the new technology respects the human being* (anthropological moment)*; and the elaboration of the ethical assessment through the examination and evaluation of the use of the specific technology* (deliberative moment)*.

## 3. Results

### 3.1. Disease Epidemiology

IDA, the most common cause of anaemia in the world, is a curable but frequently neglected disease [19] and is the main extraintestinal manifestation affecting IBD patients.  Table 1 reports the summary of studies retrieved from the relevant systematic reviews and meta-analyses. The reported prevalence of anaemia in patients with IBD is approximately 25%, with marked differences between published studies, ranging from 9% to 73% [20–22]. A recent systematic review [23], based on individual patient data from 8 European countries, reported the influence of several patient characteristics on the prevalence of anaemia in IBD and found it to be higher in men aged under 30 or over 65 years, but equal in all ages in women. In Italy specifically, the prevalence of anaemia in IBD was reported to be approximately 37%, with severe cases reported in 8.3% of the population [24].

The discrepancy in epidemiological figures may be also caused by differences in the definition of anaemia used and in the patient population (inpatient versus outpatient). The higher prevalence of anaemia in hospitalized patients indicates that anaemia could be considered as a trigger for hospitalization [22]. For instance, recent studies calculated mean prevalence of 20% among outpatients and 68% among hospitalized patients [25].

### 3.2. Efficacy and Safety of IV Iron

The symptoms of IBD can be quite severe and the condition is due to chronic intestinal blood loss from inflamed intestinal mucosa combined with the impaired absorption of iron. Almost 90% of ingested iron cannot be absorbed in IBD patients which can lead to gastrointestinal side effects (nausea, diarrhoea, constipation, and abdominal pain). Also, large amounts of iron in the lumen of the gastrointestinal tract can result in oxidative stress and inflammation in the gut [7] which may potentially lead to reactivation of IBD [21]. This can result in lower patient compliance and therefore limited effectiveness of the oral administration method [26].

Although some evidence is available regarding the superior efficacy of intravenous iron preparations over oral ones [27], there is still insufficient data from RCTs comparing different IV iron formulations in an IBD population, which limits the availability of high level evidence to support the recommendations for IV treatment [28]. Nevertheless, a recently published network meta-analysis highlights that ferric carboxymaltose may provide better and faster correction of Hb and serum ferritin levels in iron-deficient patients, with good tolerability and a minimal risk of adverse events (AEs) [29]. Only one study has supported the use of ferric carboxymaltose in IBD patients, suggesting that this regimen had a significantly higher treatment response than IV iron sucrose. In relation to side effects, no significant differences (*p* = 0.413) were observed between the two treatments [30].

An evaluation of all prospective studies regarding treatment of iron deficiency anaemia in IBD patients was performed by Nielsen et al. [25]. Summarizing results from 13 trials the authors concluded that IV iron formulations were more effective than oral treatments in relation to an increase in Hb levels in cases of severe anaemia. However, in cases of mild anaemia higher ferritin, but not Hb levels, was observed.

In addition, Bonovas and colleagues [27] conducted a systematic review and meta-analysis on randomized clinical trials comparing IV and oral iron in IBD. The authors gathered data from 5 studies and included 694 IBD patients in their analyses. The results indicated a higher efficacy of IV formulations in achieving a Hb rise of ≥2.0 g/dL (OR: 1.57, 95% CI: 1.13, 2.18). Treatment discontinuation rates relating to the drugs, as well as the occurrence of gastrointestinal AEs, were lower in the IV iron groups. Serious AEs, although more frequently reported among patients receiving IV iron, were judged as unrelated or unlikely to be related to the study medication, without the conclusion of any causal relationship.

Another study from 2013 [31] reported the safety of IV therapy for correcting anaemia in IBD, highlighting that IV formulations showed no increase in serious AEs (anaphylaxis, AEs requiring hospitalization, and AEs regarded by the authors as serious). They were also associated with a decrease in AEs that required discontinuation of treatment (RR 0.13, 95% CI 0.05–0.38) and in gastrointestinal AEs, such as abdominal pain, diarrhoea, and vomiting (RR 0.25, 95% CI 0.06–0.95).

Finally, the study by Avni et al. [32] evaluated the use of IV iron formulations for any clinical indication and reached similar findings in a much larger patient cohort.

### 3.3. Organizational Aspects

So far in Italy, different ferric (3+) formulations have been used in clinical practice, which differ in their pharmacokinetic properties and safety profiles: ferric gluconate, iron sucrose, and ferric carboxymaltose. The characteristics of these products are specified in Table 2.

The evaluation of the Summary of Product Characteristics revealed that, among three IV formulations currently available in Italy, ferric carboxymaltose has the most convenient profile in terms of time necessary to achieve a maximum single dose, as well as number of sessions and cumulative time needed to infuse the prescribed dose (Table 2). For instance, ferric gluconate, typically administered as “a very slow” infusion (not less than 50′) in a maximum dose of 125 mg, requires multiple outpatient visits and repeated intravenous access for patients to receive the standard therapeutic course of 1,000 mg of elemental iron. On the other hand, ferric carboxymaltose permits an administration of a large replenishment dose (maximum quantity of 1,000 mg) over a short infusion period (15′) and would usually allow iron repletion in one or two sessions [28, 33, 34]. These points indicate an obvious advantage of ferric carboxymaltose treatment that may carry a positive influence on patient compliance to therapy.

In addition, this issue would have a high impact on the organizational aspects of the institution itself, since it would free up a substantial number of armchairs and nursing units and allow treatment of a higher number of patients.

However, the literature review revealed some conflicting theoretical considerations regarding the length of the infusion. In particular, Bhandari considered the “multitasking” of the staff, emphasizing that the staff should be solely devoted to a single patient only for each infusion. The author assumed that, for a short infusion (e.g., 30 minutes), the nurse is dedicated to the patient for the entire duration of the treatment; for an infusion of about 60 minutes, the nurse spends around 50% of her time with the patient, while for prolonged infusions the nurse spends about 33% of her time with the patient (with the exception of the test dose in which the nursing observation may reach 100%) [34]. The work of Fragoulakis et al. [35] summarized expert opinions and technical specifications and concluded that patients receiving iron infusions should be kept under close clinical observation by a doctor and a nurse. These assumptions were useful in determining the plausibility range of our economic analyses.

### 3.4. Economic Evaluation

It is estimated that, during a one-year period, the “A. Gemelli” Foundation Polyclinic treats 559 “standard” ambulatory patients with intravenous iron therapy (1000 mg of iron). The internal medicine and gastroenterology departments delivered the largest number of iron infusions, followed by haematology and oncology. Among the patients referred to the internal medicine and gastroenterology clinics receiving intravenous iron treatment, patients with IBD represented 50–60% of the total.

The base model of the cost-minimization analysis is presented in Table 3. The ferric carboxymaltose represents the cheaper alternative and would, in the baseline scenario, allow a saving of between 263.40 and 87.10 € compared to 1 or 2 vials of ferric gluconate per session, respectively. This advantage of ferric carboxymaltose is mainly due to the significant reduction in personnel costs (between 10 and 20 times less), followed by the reduction of the cost for the use and removal of disposable materials (15–25 times less). These benefits largely balance the higher cost of the drug (more than 20 times), even without considering that due to the higher cost, the Lazio region, like most Italian regions, places this drug in a category which is fully refunded by the Local Health Authority.

Sensitivity analyses considering best and worst case scenarios are reported in Table 4. Mean time of administration of the therapy varied from 0.41 to 0.58 hours for both therapeutic strengths of ferric carboxymaltose. In addition, the range of total costs associated with ferric carboxymaltose varied from € 110.41–€ 300.61 to € 67.78–€ 165.06 for 1000 mg or 500 mg of the drug, respectively. In contrast, administration time of ferric gluconate ranged from 7,36 to 38,72 hours, according to the different strengths of the therapy and the number of sessions required. For 1000 mg of therapy, the total cost varied from 439,87 to 1,063.31 € for the 1 vial per session option and from 239.57 to 557.08 € for the two vials per session option. Similarly, the 500 mg dose produced costs of € 233.75–€ 545.50 for the 1 vial per session option and € 133.61–€ 292.36 for the 2 vials per session option.

### 3.5. Ethical Evaluation

Based on RCTs, IV iron treatment has a favourable risk-benefit profile. Particularly, compared to other treatments, ferric carboxymaltose allows a higher (1000 mg) and faster (15′) maximum dose administration as a single infusion. Furthermore, as ferric carboxymaltose provides a fast and effective response, it is generally better tolerated by patients, has fewer side effects and a lower discontinuation rate, and can be presumed to improve the patient's quality of life. Nevertheless, before starting an IV iron treatment with ferric carboxymaltose, an adequate communication process about possible risks and benefits, method of administration, and available alternatives is required to guarantee patient autonomy. Due to the unfortunately limited resources available for healthcare, it is necessary to identify these criteria and rationalize them ensuring that justice and equity are not compromised. In the person-centred ethics, the patient is the measure for decision-making and his or her health/life protection is the fundamental value. The cost-minimization analysis showed that the higher cost of ferric carboxymaltose treatment is balanced by the reduction of costs related to individual patient management, mainly due to the shorter infusion time and fewer number of sessions.

In conclusion, current evidence justifies a positive ethical evaluation of the use of ferric carboxymaltose, however; further studies are needed to address the safety profile in the postmarketing phase.

## 4. Discussion

The aim of this paper was to perform a thorough analysis of epidemiological, economical, organizational, and ethical implications of the introduction of ferric carboxymaltose into the outpatient context of the Policlinico Universitario “Agostino Gemelli”, Rome, Italy. We were particularly interested in one gastroenterology department specifically dedicated to IBD patients. The multilevel approach of our HTA report supported a positive conclusion regarding the drug.

Our literature review showed that the reported prevalence of anaemia in patients with IBD varies from 10 to 73% for CD and from 9 to 67% for UC [23]. IDA occurs frequently as an extraintestinal complication in IBD patients and is associated with an aggressive and disabling disease, requiring higher healthcare utilization. It can be considered as a significant predictor of increased risk of hospitalization and even increased patient mortality [5].

Data regarding the efficacy of IV iron formulations was mainly drawn from meta-analyses and RCTs which encompassed a placebo or oral iron supplementation group and were not head-to-head studies [28, 29, 31, 32]. Despite this limiting factor, the literature indicates that intravenous iron preparations are safe and effective, with the most recent evidence [29] suggesting a favourable effect of ferric carboxymaltose for a better and faster correction of Hb and serum ferritin levels in iron-deficient patients.

Several scientific papers have assessed the economic implications of the available therapeutic options for the IV treatment of anaemia, from the perspective of the National Health Systems (28–30). For example, Gutzwiller et al. demonstrated that iron repletion using IV ferric carboxymaltose in patients with heart failure is cost effective from the UK payers' perspective (National Health Service) [36], while according to Fragoulakis et al. ferric carboxymaltose may even represent a cost-saving option, especially when administered in an outpatient setting [35]. Furthermore, one budget-impact analysis focused on the replacement of ferric saccharate (standard treatment) with the new ferric carboxymaltose within the Swiss Health System. After performing real-life data analyses, the authors estimated a total saving of about CHF 22 million to 31 million (equivalent to 20–28 million euro) related to use of ferric carboxymaltose, mainly driven by savings in staff costs (due to the difference in the duration and frequency of administration of the regimen) [37].

Although the perspective of the NHS generates the highest interest, the current economic environment with exponential increases in healthcare costs and limited resources shifts the focus of HTA evaluation to the perspective of service providers [38]. The literature has demonstrated several examples of studies conducted to analyse the economic implications of different IV treatments of anaemia in a hospital setting. One recent study, conducted in a hospital outpatient gastroenterology in Spain, showed a reduction in direct annual costs of about 30 euro per patient in the case of the use of ferric carboxymaltose compared to ferric saccharate, a sum that rose to 67 euro per year after also considering nonhospital direct costs (patients' movements and loss of productivity for the patient and accompanying persons) [39].

The evidence from the above-mentioned studies, which were undertaken with similar purposes, but in different countries and from different perspectives, is in line with our hospital-based cost-analysis. The latter implicated that, due to the reduction in personnel costs and expenses related to the use and removal of disposable materials, ferric carboxymaltose may generate a saving for the hospital of between 263.40 and 87.10 € compared to 1 or 2 vials of ferric gluconate per session, respectively. Considering all possible scenarios defined in the sensitivity analysis, ferric carboxymaltose was found to result in the lowest cost for the hospital.

Based on the number of patients that need to be treated each year and thus on the number of hours required for the infusion, and if all the patients requiring intravenous iron supplementation are to be treated with ferric carboxymaltose, Fondazione Policlinico Gemelli could free up between 8 and 16 armchairs and between 2 and 4 nursing units. In this scenario, having fully satisfied the ambulatory request related to patients with anaemia, these resources could be devoted to other activities, perhaps giving priority to those with the greatest waiting list urgency. In contrast, considering the chronic shortage of staff, particularly nurses, it would be possible to adapt a partial redeployment of nursing units to the clinics in the most need. Another possibility may be to reinvest these resources to increase the number of patients treated by the infusion centre, which would make it more feasible to meet the treatment needs of anaemia patients, ensuring better care for a frail IBD population who are commonly “undertreated” for comorbidities such as anaemia.

Currently, there are no economic evaluations available regarding the introduction of ferric carboxymaltose for IDA treatment in the Italian context. This Hospital-Based Health Technology Assessment collected, combined, and synthetized all the available data which are essential to support the sustainable introduction of a new drug. Considering the high prevalence of anaemia, especially in IBD patients, and the recent possibility of choosing different treatment options, this analysis is highly desirable and might have a valuable role in guiding treatment decisions in the Italian outpatient setting.

## 5. Conclusions

Iron deficiency anaemia is a common and curable disease that is frequently neglected. IDA represents the most frequent extraintestinal complication in IBD patients and it is associated with aggressive and disabling disease requiring higher healthcare utilization. Timely and adequate IV iron treatment is strongly indicated in this population. Despite all currently available intravenous iron preparations being safe and effective, ferric carboxymaltose seems to have a more favourable risk-benefit profile. However, the higher costs compared to currently available treatments in outpatient settings in Italy could prevent its use in real life. Nevertheless, according to this Hospital-Based Health Technology Assessment, the introduction of ferric carboxymaltose presents several advantages which allow a positive organizational, economical, and ethical evaluation. In particular, the reduction of costs related to individual patient management with ferric carboxymaltose can compensate for the higher cost of the drug, allowing health facilities to offer a higher quality performance, especially in current times of limited economic and organic resources. In contrast, lower number of infusions and reduced access to health facilities represent a clear benefit for patients, especially for those with multiple chronic conditions, with reduced availability of peripheral venous access and/or with difficulties to reach the health facilities.

## Figures and Tables

**Table 1:** Summary of individual studies on prevalence of anaemia in IBD, retrieved from recent literature reviews^*∗*^.

Author	Year	Country	Patients (*n*)	Prevalence of anaemia (%)		
				IBD	CD	UC
Koutroubakis et al.	2016	USA	1,821	50.1	53.3 (574/1,077)	44.7 (333/744)
Hoivik et al.	2014	Norway	756	29.1	48.8 (116/237)	20.2 (104/519)
Sjoberg et al.	2014	Sweden	749	30	42 (107/254	24 (119/495)
Toruner et al.	2014	Turkey	398	45.11	24 (45/186)	22 (47/212)
Rejler et al.	2012	Sweden	485	6	9	5
Bager et al.	2011	Denmark, Norway, Sweden	429	17	23.6 (60/254)	7.4 (13/175)
Bergamaschi et al.	2010	Italy	263	36.96	43 (71/165)	34 (33/98)
Blumenstein et al.	2011/2008	Germany	636	18.87	17 (57/336)	21 (63/300)
Goodhand et al.	2011	UK	124	40	48	45
Oustamanolakis et al.	2011	Greece	100	42	42 (20/48)	41 (21/51)
Romberg-Camps et al.	2010	Netherlands	707	9.2	11.5	7.3
Voegtlin et al.	2010	Switzerland	241	19.92	17 (23/137)	24 (25/104)
De la Morena and Gisbert	2009	Spain	253	24.11	32 (43/136)	15 (18/117)
Vijverman et al.	2006	Belgium	170	24.7	/	/
Ebinger et al.	2004	Germany	599	4.8	3.2 (13/390)	6.2 (13/209)
Ershler et al.	2005	USA	7200	13	/	/
Lakatos et al.	2003	Hungary	254	26.5	35.8	25
Revel-Vilke et al.	2000	Israel	63	41.3	/	/
Schreiber et al.	1996	Germany	676	31.7	26 (88/334)	37 (126/342)
Gasche et al.	1994	Austria	49	/	33 (16/49)	/
Horina et al.	1993	Austria	85	33	/	/

^*∗*^Modified from Filmann et al. [23], Stein et al. [21], and Kulnigg and Gasche [22].

**Table 2:** Intravenous iron formulations available in Italy.

Active ingredient	Ferric gluconate	Ferric carboxymaltose	Iron sucrose
Pharmaceutical form (volume in ml)	po/IV vial (5)	IV vial (10)	IV vial (5)

Amount of elemental iron/vial (mg)	62.5	500	100

Indications	Treatment of iron deficiency anaemia in patients with lack of response to oral iron	Treatment of iron deficiency anaemia in patients with known intolerance or lack of response to oral iron	Iron deficiency: in case of a clinical need for rapid release of iron from reserves: (i) in case of drug intolerance or noncompliant patients; (ii) in case of IBD patients in active phase of disease, when oral iron treatment is not efficient

Maximum dose that can be administered as a single infusion (mg)	From 62.5 to 125	1000	200

Cumulative dose prescribed /patient (based on the most commonly calculated iron needs) (mg)	1000	1000	1000

Time necessary to reach maximum single dose (min)	“Very slowly” (not less than 50)	15 (for a maximum quantity of 1000 mg)	30 (for a maximum quantity of 200 mg)

Number of sessions/visits needed to instil the prescribed dose of 1000 mg (RCP)	16 for a single dosage of 62,5 mg and 8 for a single dosage of 125 mg	1	5

Cumulative time necessary to instil the prescribed dose of 1000 mg (minutes)	(i) 800 when applying “very slowly” (50 per every infusion of 62.5 mg) (ii) 400 for dosage of 125 mg	15	150

**Table 3:** Cost analysis: base model.

Base model	Ferric carboxymaltose 1000 Mg	Ferric gluconate 1000 Mg (1 vial per session)	Ferric gluconate 1000 Mg (2 vials per session)
Number of exams per year	559	559	559
Administration time (hours)	0.41	14.72	7.36
Cost of labour (€)	24.34	405.49	216.56
Medication and instrumentation costs (€)	182.81	36.81	25.44
Total costs (€)	207.15	442.30	242.00
Regional reimbursement (€)	108.91	80.66	56.66
Reimbursement costs (€)	−98.24	−361.64	−185.34

**Table 4:** Cost analysis: summary of the sensitivity analysis—best and worst case.

Total iron dose for patient	Variables	Ferric carboxymaltose	Ferric gluconate (1 vial per session)	Ferric gluconate (2 vials per session)
1000 Mg	Administration time (hours)	0.41–0.58	14.72–38.72	7.36–19.36
	Labor cost (€)	24.34–28.70	405.49–1017.47	216.56–522.55
	Medication and instrumentation costs (€)	86.07–271.91	34.38–45.9	23.01–34.53
	Total costs (€)	110.41–300.61	439.87–1.063.31	239.57–557.08
	Regional reimbursement (€)	108.91	80.66	56.66
	Reimbursement cost (€) Variation best-worst case	−1.50–−191.70	−359.21–−982.65	−182.91–−500.42

500 Mg	Administration time (hours)	0.41–0.58	7.36–19.36	3.68–9.68
	Labor cost (€)	24.34–28.70	216.56–522.55	122.10 –275.09
	Medication and instrumentation costs (€)	43.44–136.36	26.54–33.26	15.17–21.89
	Total costs (€)	67.78–165.64	233.75–545.50	133.61–292.36
	Regional reimbursement (€)	66.29	56.66	44.66
	Reimbursement cost (€) Variation best-worst case	−1.49–−99.35	−177.09–−488.84	−88.95–−247.70

## References

[1] Dignass A. U., Gasche C., Bettenworth D. (2015). European consensus on the diagnosis and management of iron deficiency and anaemia in inflammatory bowel diseases. *Journal of Crohn's & colitis*.

[2] Dudkowiak R., Neubauer K., Poniewierka E. (2013). Hepcidin and its role in inflammatory bowel disease. *Advances in Clinical and Experimental Medicine*.

[3] Lomer M. C. E. (2011). Dietary and nutritional considerations for inflammatory bowel disease. *The Proceedings of the Nutrition Society*.

[4] Voudoukis E., Karmiris K., Oustamanolakis P. (2013). Association between thrombocytosis and iron deficiency anemia in inflammatory bowel disease. *European Journal of Gastroenterology and Hepatology*.

[5] Antunes C. V., Hallack Neto A. E., Nascimento C. R. (2015). Anemia in Inflammatory bowel disease outpatients: prevalence, risk factors, and etiology. *BioMed Research International*.

[6] Koutroubakis I. E., Ramos-Rivers C., Regueiro M. (2015). Persistent or recurrent anemia is associated with severe and disabling inflammatory bowel disease. *Clinical Gastroenterology and Hepatology*.

[7] Lugg S., Beal F., Nightingale P., Bhala N., Iqbal T. (2014). Iron treatment and inflammatory bowel disease: What happens in real practice?. *Journal of Crohn's and Colitis*.

[8] Bayraktar U. D., Bayraktar S. (2010). Treatment of iron deficiency anemia associated with gastrointestinal tract diseases. *World Journal of Gastroenterology*.

[9] Guagnozzi D., Lucendo A. J. (2014). Anemia in inflammatory bowel disease: a neglected issue with relevant effects. *World Journal of Gastroenterology*.

[10] Kulnigg S., Stoinov S., Simanenkov V. (2008). A novel intravenous iron formulation for treatment of anemia in inflammatory bowel disease: the ferric carboxymaltose (FERINJECT) randomized controlled trial. *The American Journal of Gastroenterology*.

[11] Friedrisch J. R., Cancado R. D. (2015). Intravenous ferric carboxymaltose for the treatment of iron deficiency anemia. *Revista Brasileira de Hematologia e Hemoterapia*.

[12] Shander A., Goodnough L. T., Javidroozi M. (2014). Iron deficiency anemia-bridging the knowledge and practice gap. *Transfusion Medicine Reviews*.

[13] Battista R. N. (2006). Expanding the scientific basis of health technology assessment: A research agenda for the next decade. *International Journal of Technology Assessment in Health Care*.

[14] Taylor R. S., Drummond M. F., Salkeld G., Sullivan S. D. (2004). Inclusion of cost effectiveness in licensing requirements of new drugs: The fourth hurdle. *British Medical Journal*.

[15] Specchia M. L., De Waure C., Gualano M. R. (2014). Health technology assessment of belimumab: a new monoclonal antibody for the treatment of systemic lupus erythematosus. *BioMed Research International*.

[16] de Waure C., Specchia M. L., Cadeddu C. (2014). The prevention of postmenopausal osteoporotic fractures: results of the health technology assessment of a new antiosteoporotic drug. *BioMed Research International*.

[17] farmadati Farmadati italia - banca dati del farmaco, parafarmaco e dispositivo medico. http://www.farmadati.it/.

[18] APAT: Agenzia per la protezione dell’ambiente e per i servizi tecnici (2008). *Valutazioni Quali-Quantitative Sulla Produzione E Gestione Dei Rifiuti Speciali Sanitari*.

[19] Miller J. L. (2013). Iron deficiency anemia: A common and curable disease. *Cold Spring Harbor Perspectives in Medicine*.

[20] Wilson A., Reyes E., Ofman J. (2004). Prevalence and outcomes of anemia in inflammatory bowel disease: a systematic review of the literature. *The American Journal of Medicine*.

[21] Stein J., Hartmann F., Dignass A. U. (2010). Diagnosis and management of iron deficiency anemia in patients with IBD. *Nature Reviews Gastroenterology and Hepatology*.

[22] Kulnigg S., Gasche C. (2006). Systematic review: managing anaemia in Crohn's disease. *Alimentary Pharmacology and Therapeutics*.

[23] Filmann N., Rey J., Schneeweiss S. (2014). Prevalence of anemia in inflammatory bowel diseases in European countries: a systematic review and individual patient data meta-analysis. *Inflammatory Bowel Diseases*.

[24] Bergamaschi G., di Sabatino A., Albertini R. (2010). Prevalence and pathogenesis of anemia in inflammatory bowel disease. Influence of anti-tumor necrosis factor-*α* treatment. *Haematologica*.

[25] Nielsen O. H., Ainsworth M., Coskun M., Weiss G. (2015). Management of Iron-Deficiency Anemia in Inflammatory Bowel Disease: a Systematic Review. *Medicine (United States)*.

[26] Lindgren S., Wikman O., Befrits R. (2009). Intravenous iron sucrose is superior to oral iron sulphate for correcting anaemia and restoring iron stores in IBD patients: a randomized, controlled, evaluator-blind, multicentre study. *Scandinavian Journal of Gastroenterology*.

[27] Bonovas S., Fiorino G., Allocca M. (2016). Intravenous versus oral iron for the treatment of anemia in inflammatory bowel disease: a systematic review and meta-analysis of randomized controlled trials. *Medicine (Baltimore)*.

[28] Moore R. A., Gaskell H., Rose P., Allan J. (2011). Meta-analysis of efficacy and safety of intravenous ferric carboxymaltose (Ferinject) from clinical trial reports and published trial data. *BMC Blood Disorders*.

[29] Rognoni C., Venturini S., Meregaglia M., Marmifero M., Tarricone R. (2016). Efficacy and Safety of Ferric Carboxymaltose and Other Formulations in Iron-Deficient Patients: A Systematic Review and Network Meta-analysis of Randomised Controlled Trials. *Clinical Drug Investigation*.

[30] Evstatiev R., Marteau P., Iqbal T. (2011). FERGIcor, a randomized controlled trial on ferric carboxymaltose for iron deficiency anemia in inflammatory bowel disease. *Gastroenterology*.

[31] Avni T., Bieber A., Grossman A., Green H., Leibovici L., Gafter-Gvili A. (2015). The safety of intravenous iron preparations: systematic review and meta-analysis. *Mayo Clinic Proceedings*.

[32] Avni T., Bieber A., Steinmetz T., Leibovici L., Gafter-Gvili A. (2013). Treatment of anemia in inflammatory bowel disease- systematic review and meta-analysis. *PLoS ONE*.

[33] Lyseng-Williamson K. A., Keating G. M. (2009). Ferric Carboxymaltose. *Drugs*.

[34] Bhandari S. (2011). Update of a comparative analysis of cost minimization following the introduction of newly available intravenous iron therapies in hospital practice. *Therapeutics and Clinical Risk Management*.

[35] Fragoulakis V., Kourlaba G., Goumenos D., Konstantoulakis M., Maniadakis N. (2012). Economic evaluation of intravenous iron treatments in the management of anemia patients in Greece. *ClinicoEconomics and Outcomes Research*.

[36] Gutzwiller F. S., Schwenkglenks M., Blank P. R. (2012). Health economic assessment of ferric carboxymaltose in patients with iron deficiency and chronic heart failure based on the FAIR-HF trial: An analysis for the UK. *European Journal of Heart Failure*.

[37] Brock E., Braunhofer P., Troxler J., Schneider H. (2013). Budget impact of parenteral iron treatment of iron deficiency: Methodological issues raised by using real-life data. *European Journal of Health Economics*.

[38] Volpe M., Scaldaferri F., Ojetti V., Poscia A. (2013). Breath tests sustainability in hospital settings: cost analysis and reimbursement in the Italian National Health System. *European Review for Medical and Pharmacological Sciences*.

[39] Calvet X., Ruíz M. À., Dosal A. (2012). Cost-minimization analysis favours intravenous ferric carboxymaltose over ferric sucrose for the ambulatory treatment of severe iron deficiency. *PLoS ONE*.

